# Rowan Berries: A Potential Source for Green Synthesis of Extremely Monodisperse Gold and Silver Nanoparticles and Their Antimicrobial Property

**DOI:** 10.3390/pharmaceutics14010082

**Published:** 2021-12-29

**Authors:** Priyanka Singh, Ivan Mijakovic

**Affiliations:** 1 The Novo Nordisk Foundation, Center for Biosustainability, Technical University of Denmark, DK-2800 Kogens Lyngby, Denmark; 2 Systems and Synthetic Biology Division, Department of Biology and Biological Engineering, Chalmers University of Technology, SE-412 96 Gothenburg, Sweden

**Keywords:** gold nanoparticles, silver nanoparticles, *Sorbus aucuparia*, rowanberries, monodisperse, stable, antimicrobial activity

## Abstract

Rowanberries (*Sorbus aucuparia*) are omnipresent in Europe. The medicinal importance of rowanberries is widely known and corresponds to the active ingredients present in the fruits, mainly polyphenols, carotenoids, and organic acids. In the current study, we explored rowanberries for the reduction of gold and silver salts into nanoparticles. Rowanberries-mediated gold nanoparticles (RB-AuNPs) formed within 5 s at room temperature, and silver nanoparticles (RB-AgNPs) formed in 20 min at 90 °C. The produced nanoparticles were thoroughly characterized by UV-Vis spectroscopy, scanning electron microscopy (SEM), energy dispersive X-ray (EDX), transmission electron microscopy (TEM), dynamic light scattering (DLS), single-particle inductively coupled plasma–mass spectrometry (sp-ICP-MS), thermogravimetric analysis (TGA), Fourier transform-infrared spectroscopy (FT-IR) and matrix-assisted laser desorption/ionization time of flight mass spectrometry (MALDI-TOF). The characterization confirmed that the nanoparticles are highly monodisperse, spherical, stable over long periods, and exhibit a high negative zeta potential values. The produced RB-AuNPs and RB-AgNPs were 90–100 nm and 20–30 nm in size with a thick biological corona layer surrounding them, providing extreme stability but lowering the antimicrobial activity. The antimicrobials study of RB-AgNPs revealed that the nanoparticles have antimicrobial potential with an MBC value of 100 µg/mL against *P. aeruginosa* and 200 µg/mL against *E. coli*.

## 1. Introduction

Among all the developed methodologies for nanoparticles production, the green methodologies are considered facile, non-toxic, eco-friendly, and economical. Various green resources are available for this task, including bacteria, fungi, yeast, plants parts (roots, leaves, flowers, and fruits). This implies that the green resources contain numerous biological components, such as metabolites, sugars, proteins, polysaccharides, amino acids, which play an important role in reducing and stabilizing nanoparticles [1,2]. In addition to making stable nanoparticles, these biological components improve the synthesis rate and provide biocompatibility. Comparatively, the physio-chemical approaches are cost demanding, slow, produce hazardous byproducts, and most importantly, add toxic components on nanoparticle’s surfaces, limiting their medicinal applications. This motivates the discovery of novel green and efficient techniques for nanoparticles production [3]. Especially, the use of medicinal plants is becoming more popular because this results in the attachment of medicinal components to the biological corona, which surrounds the nanoparticles. This biological corona later helps to improve the efficacy of nanoparticles in various medical applications or provides long-term stability, sometimes both [4].

Rowanberries, also known as mountain ashes, are mainly shrubs or trees and belong to the *Sorbus* genus of the rose family, *Rosaceae*. Rowanberries contain many beneficial components used for medicinal purposes. Historically, rowanberries have been utilized as an anti-inflammatory, antidiarrheal, diuretic, and vasodilator agents. In some nations, rowanberries have also been used to treat intestinal blockages, liver and gallbladder problems. Rowanberries have long been used in folk medicine as an appetite-improving agent and an excellent source of vitamins—ascorbic acid (vitamin C), gargle juice to treat hoarseness, a gentle laxative treatment, and a treatment for rheumatism and renal ailments. The tea, syrup, jelly, or alcoholic tincture of rowan berries have been used to treat fever, infections, colds, flu, rheumatism, and gout. In addition, rowanberries contain sorbitol which is suitable as a sweetener for diabetics [5]. The medicinal use of rowanberries is based on the significant amounts of phytochemicals, such as phenolic acids, vitamins, carotenoids, and important minerals, such as iron, copper, zinc, potassium, and magnesium [6]. Chlorogenic and neochlorogenic acids are the main phenolic acids that constitute 56–80% of the total phenolics in rowanberries [7]. Other main phenolic acids (catechin, epicatechin, ferulic acid methyl ester, procyanidin B1), flavonols (quercetin, isoquercetin, hyperoside, rutin, catechin, epicatechin), anthocyanins (mainly cyanidin or pelargonidin glycosides), and proanthocyanidins are found in the aqueous extract of rowanberries [8]. Some other well-known phenolic acids and their derivatives found in traces in the rowanberries are cinnamic, vanillic, p-coumaric, caffeic acid, and benzoic acids [9]. The polyphenolics found in rowanberries have revealed strong antioxidant, antidiabetic, anti-hyperlipidemic, anti-inflammatory, anticancer, antimicrobial, anti-periodontal, and anti-osteoarthritis effects [10]. They also possess vasoprotective, neuroprotective, cardioprotective, hepatoprotective properties, and cyclooxygenase–2 inhibitory activities [10]. In addition, many studies have reported quercetin, sexangularetin, and kaempferol glycosides in the berries. Marcotullio et al. demonstrated that rowanberries contain mainly polyphenols 1.34–1.47 g/100 g, carotenoids 21.65 mg/100 g, and various organic acids, such as malic and citric, and succinic [8]. The main carotenoids in rowanberries are zeaxanthin, β-cryptoxanthin, and all-*trans*-β-carotene [10]. Parasorbic acid and the cyanogenic glycoside prunasin are two toxic components found in the pomace of rowanberries, which are also found in the seeds. Parasorbic acid in excessive amounts causes indigestion and kidney damage. However, heat treatment or freezing transforms parasorbic acid into harmless sorbic acid. The cyanogenic glycoside prunasin can release hydrogen cyanide, which can induce respiratory failure and even death if it exceeds the 2–3 mg/L limit [11]. Therefore, seeds are toxic and are mostly considered waste for any purpose. In the current study, we used whole fruits, including seeds, to extract the biomolecules and efficiently produce gold and silver nanoparticles.

Biogenic gold and silver nanoparticles are documented as biocompatible, stable, and monodisperse [12]. Biogenic nanoparticles have been applied for many medical benefits ranging from antimicrobial, anticancer, anti-inflammatory, antioxidant, and other optical applications [13,14,15,16]. These applications are possible due to the tunable size and shape-dependent properties of nanoparticles [1]. Following the importance of green nanoparticles [17], we used rowanberries’ aqueous extract to form stable, monodisperse, and spherical gold and silver nanoparticles. In addition to thorough analytical characterizations, we explored the rowanberries mediated silver nanoparticles (RB-AgNPs) for antimicrobial application against two Gram-negative pathogens: *P. aeruginosa* and *E. coli*.

## 2. Materials and Methods

### 2.1. Materials

Analytical grade gold(III) chloride trihydrate (HAuCl_4_·3H_2_O) and silver nitrate (AgNO_3_) were purchased from Sigma–Aldrich Chemicals, (St Louis, MO, USA). Fresh berries were harvested from the tree, washed twice with distilled water to remove any dust or unwanted components, and air-dried overnight. Ten grams of berries were added to a sterile flask containing 90 mL of distilled water and autoclaved for 20 min at 100 °C. After autoclaving, the mixture was filtered to remove the particulates completely. Further purification was done by centrifugation at 8000 rpm for 3 min, which helped to eliminate fine suspended particles. This liquid was considered as a stock solution and used for nanoparticle production in different dilutions. The aqueous extract was diluted with different ratios of water and referred as a synthesis medium (SM) [2].

### 2.2. Green Synthesis of RB-AuNPs and RB-AgNPs

For the nanoparticle’s synthesis, the optimized concentration of gold salt (HAuCl_4_·3H_2_O) and silver salt (AgNO_3_) was added to the SM, which contains an aqueous extract of rowanberries and water in an optimum ratio. The SM was further incubated at a defined time and temperature. The nanoparticles production was first examined by the color change in the SM, following spectral analysis [18]. Once the nanoparticles were formed, they were purified by centrifugation at 2000 rpm for 5 min, which allowed the large particulates to be removed, followed by centrifugation at 14,000 rpm for 15 min to collect the fine nanoparticles. In addition, the nanoparticles were washed thrice with distilled water to remove the unconverted metal ions or other constituents [19]. Finally, the nanoparticles were collected in a pellet and resuspended in water, used for analytical characterization and application. For thermogravimetric analysis (TGA) and Fourier Transform-Infrared Spectroscopy (FT-IR), nanoparticles were air-dried to form a pellet.

### 2.3. Analytical Characterization of RB-AuNPs and RB-AgNPs

#### 2.3.1. UV-Vis Study

The reduction of gold and silver ions to RB-AuNPs and RB-AgNPs was initially monitored via visible inspection and then by scanning the SM in UV-Vis spectroscopy at a specific interval. The UV-Vis spectrum was obtained using a 6705 UV-Vis spectrophotometer, JENWAY (Cole-Parmer Ltd., Stone, UK), by scanning 1 mL of the SM in the range of 300–700 nm. The optimization studies for RB-AuNPs and RB-AgNPs production were also conducted using visible and UV-Vis spectrum analysis [20].

#### 2.3.2. Single-Particle Inductively Coupled Plasma-Mass Spectrometry (sp-ICP-MS)

sp-ICP-MS (NexION 350D; PerkinElmer Inc., Waltham, MA, USA) was performed to know the concentration of produced RB-AuNPs and RB-AgNPs. The stability of nanoparticles was examined by using the purified RB-AuNPs and RB-AgNPs solutions and keeping them for different times, temperatures, and indifferent bacteriological media, such as tryptic soy broth (TSB) and Luria broth (LB). The results were taken by visible inspection of which pictures are shown, UV-Vis, and sp-ICP-MS analysis before and after the defined period [21].

#### 2.3.3. Thermogravimetric Analysis (TGA)

TGA (TA Instruments, New Castle, DE, USA) was performed to check the temperature stability of nanoparticles. For analysis, the dried pellet of nanoparticles was placed in an alumina pan and heated from 20 to 700 °C at a ramping time of 10 °C/min.

#### 2.3.4. Scanning Electron Microscopy (SEM), Energy Dispersive X-ray (EDX), and Elemental Mapping

SEM with EDX examination and elemental mapping was performed to study the RB-AuNPs and RB-AgNPs morphology and elemental composition. EDX analysis setup was coupled with the SEM instrument. Sample preparation was done by dropping 5 µL of pure RB-AuNPs and RB-AgNPs solution (0.1 mg/mL) on carbon tape and air-dried at room temperature (RT) for 15 min. SEM micrographs were recorded using a Quanta FEG 200 ESEM microscope (Quorum Technologies, Hitachi High-Tech Europe GmbH, Sweden) [22,23].

#### 2.3.5. Transmission Electron Microscopy (TEM)

TEM study using FEI Tecnai T20 G2 (FEI, Hillsboro, OR, USA) was conducted to analyze the structural morphology and crystallographic information about RB-AuNPs and RB-AgNPs. The instrument was operated at an acceleration voltage of 200 kV. A sample of RB-AuNPs and RB-AgNPs samples was prepared by spotting a drop of pure nanoparticles solution suspended in water on a carbon-coated copper grid. The sample-containing grid was completely dried before analysis [22,24].

#### 2.3.6. Dynamic Light Scattering (DLS) Analysis

DLS measurements were performed to study the size distribution concerning intensity and zeta potential of pure RB-AuNPs and RB-AgNPs. Particle size measurement was executed using a Zetasizer Nano ZS, Chuo-ku Kobe-shi, Japan. The autocorrelation functions of the samples were analyzed using the Contin algorithm through the Zetasizer 7.12 software [25].

#### 2.3.7. Fourier Transform Infrared Spectroscopy (FT-IR)

RB-AuNPs and RB-AgNPs were subjected to FT-IR analysis to determine the presence of biomolecules, functional groups responsible for the reduction and capping/stabilization. The FT-IR measurements were carried out using a Nicolet iS50 (ThermoFisher Scientific, Waltham, MA, USA) by scanning the purified pellet of RB-AuNPs, RB-AgNPs, and freeze-dried rowanberries aqueous extract, (%) versus wavenumber (cm^−1^) [26].

#### 2.3.8. MALDI-TOF Mass Spectrometry

MALDI-TOF was conducted as mentioned previously [4]. Briefly, purified nanoparticles (1 μL) were loaded onto an AnchorChip^TM^ target plate (Bruker-Daltonics, Bremen, Germany), covered by 1 μL matrix solution (0.5 μg/μL 2,5—dihydroxybenzoic acid in 90% (*v*/*v*) acetonitrile, 0.1% (*v*/*v*) trifluoroacetic acid (TFA), and washed with 0.5% (*v*/*v*) TFA. All the analyses were performed by a MALDI-TOF mass spectrometer (Ultraflex II, Bruker-Daltonics, Bremen, Germany) in positive ion reflector modes with 1000 laser shots per spectrum using Flex Control v3.4. Spectra were processed by Flex Analysis v3.0 (Bruker-Daltonics, Bremen, Germany), and mass calibration was performed using protein standards (tryptic digest of β-lactoglobulin, 5 pmol/μL) [21].

### 2.4. Antibacterial Activity of RB-AgNPs

#### 2.4.1. RB-AgNPs Effects on Gram-Negative Pathogens

The antimicrobial activity of RB-AgNPs was evaluated against two Gram-negative pathogens: *Escherichia coli* UTI 89, and *Pseudomonas aeruginosa* PAO1. Both the strains were grown overnight in LB medium at 37 °C. The overnight grown cultures were diluted to approximately 1–2 × 10^5^ colony-forming units (CFU)/mL using LB medium. Then, the RB-AgNPs were added in concentrations ranging from 0.1 to 16 µg/mL. The LB medium containing respective pathogenic bacteria and RB-AgNPs were further incubated in a shake flask incubator at 37 °C, 150 rpm, for 24 h. After 24 h, the samples were analyzed by measuring the optical density (OD) at 550 nm. The MBC value was calculated as the lowest concentration of RB-AgNPs required to kill the respective bacterial strain. To determine the MBC value, 100 µL of the LB medium containing respective pathogenic bacteria and RB-AgNPs were spread on agar plates and incubated at 37 °C overnight, followed by a CFU count [21].

#### 2.4.2. Live and Dead Staining

To visualize the viable and dead cells, we used the Live/Dead BacLight Viability kit L13152 (Invitrogen, Molecular Probes, Inc., Eugene, OR, USA). Control and treated cells were stained for 20 min with a 6.0 μM SYTO 9 and 30 μM KI mixture. Fluorescence microscopic imaging of the cells was performed using a LEICA DM 4000 B (Leica Microsystems, Denmark) [20].

#### 2.4.3. SEM Analysis of Treated Cells

To evaluate the drastic effects of RB-AgNPs on individual cells, SEM analysis was carried out [25]. SEM analysis was performed by fixing the control and treated cells with 3% of glutaraldehyde overnight at 4 °C. The next day, samples were dehydrated with graded series of ethanol concentrations (40, 50, 60, 70, 80, and 90%) for 15 min and with absolute ethanol for 20 min. The dehydrated samples were placed on SEM carbon tape and left to dry at room temperature. The samples were coated with gold using a quorum coater before SEM imaging. In addition to SEM imaging, EDX and elemental mapping of RB-AgNPs treated cells was also performed to check that the killing effects are due to the action of RB-AgNPs only [23].

## 3. Results

### 3.1. Green Synthesis of RB-AuNPs and RB-AgNPs

**Figure 1:**
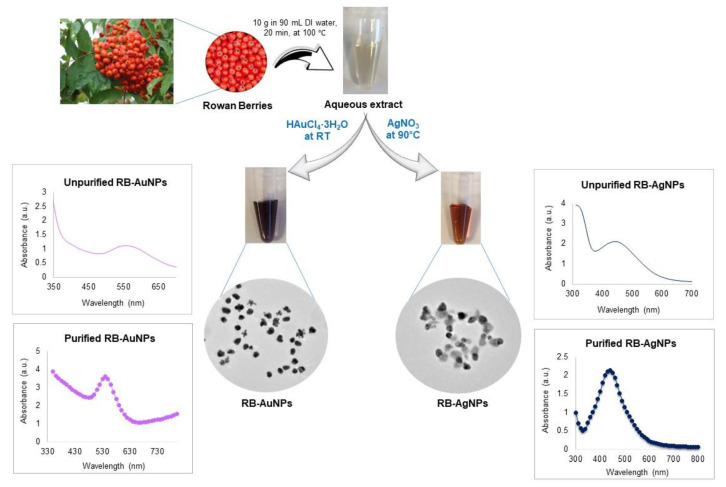
Schematic representation and UV-Vis spectra of RB-AuNPs and RB-AgNPs formation form an aqueous extract of rowanberries.

Green synthesis of RB-AuNPs and RB-AgNPs was monitored visibly and by taking spectrum with UV-Vis spectroscopy. Rowanberries extract reduced both gold and silver salt to RB-AuNPs and RB-AgNPs, respectively (Figure 1). The synthesis was confirmed visibly by a change in color of the SM, i.e., the mixture of rowanberries extract, water, and salt solution. For RB-AuNPs, the color changed to dark purple, and for RB-AgNPs, the color changed to brown from the whiteish color of rowanberries extract. This color change corresponded to the surface plasmon resonance (SPR) property of the formed nanoparticles. The synthesis was further confirmed by taking a UV-Vis spectrum in the range of 300 to 700 nm. For RB-AuNPs, a clear peak was formed in the region of 500–600 nm, and for RB-AgNPs, the maximum peak was noticed in the region of 400–500 nm [27]. The nanoparticles samples were purified by three cycles of centrifugation after DI water washing and then scanned again in UV-Vis spectroscopy. The results showed a high-intensity peak in the same region. Thus nanoparticles formed in the solution (Figure 1).

**Figure 2:**
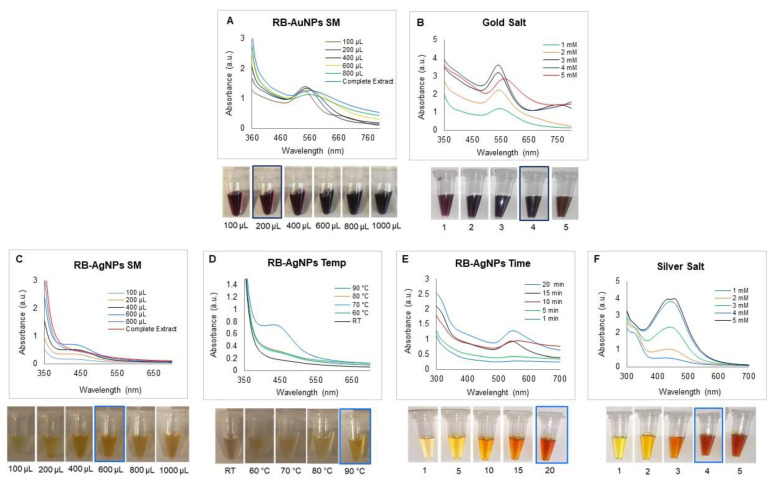
Optimization studies for RB-AuNPs and RB-AgNPs production. For RB-AuNPs production, visible picture and UV-Vis spectrum (**A**) synthesis medium (SM) optimization, (**B**), gold salt optimization. For RB-AgNPs production, visible picture and UV-Vis spectrum (**C**) synthesis medium (SM), (**D**) temperature, (**E**) synthesis time, (**F**) silver salt concentration.

The optimization studies for RB-AuNPs and RB-AgNPs were also conducted by using UV-Vis spectrum analysis and visible observations [28]. For RB-AuNPs, the optimum ratio of SM was 2:8 (Figure 2A), gold salt concentration was 4 mM (Figure 2B), and the reaction took place at room temperature within 5 s. The synthesis was so quick that the rowanberries extract showed complete color change at RT as soon as gold salt was added. For RB-AgNPs, the optimized ratio of SM for nanoparticles production was 6:4 (Figure 2C), temperature 90 °C (Figure 2D), synthesis time 20 min (Figure 2E), and at 4 mM silver salt concentration (Figure 2F).

### 3.2. Characterization of RB-AuNPs and RB-AgNPs

**Figure 3:**
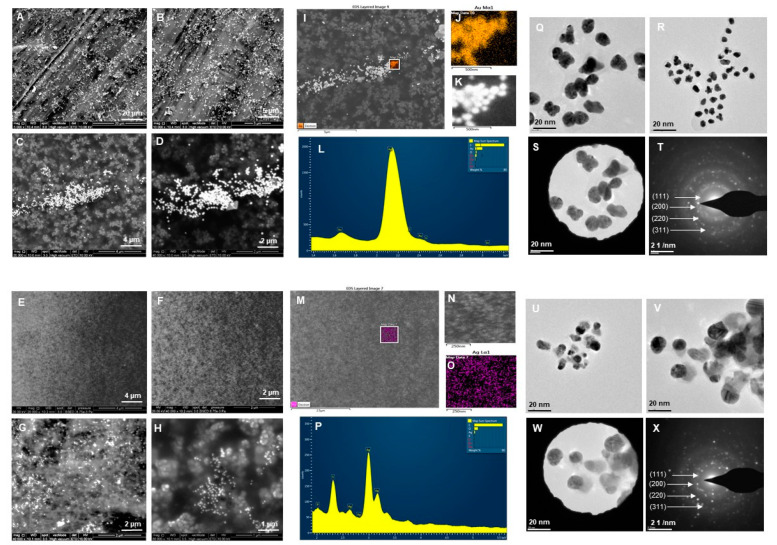
Structural analysis of RB-AuNPs and RB-AgNPs. For RB-AuNPs, (**A**–**D**) SEM images of nanoparticles at different scales, (**I**–**K**) Elemental mapping of RB-AuNPs showing scanned image of NPs with gold element distribution (orange), (**L**) EDX spectrum of the elemental mapped region showing sharp peak for gold element. (**Q**,**R**) TEM image of RB-AuNPs, (**S**,**T**) SAED pattern. For RB-AgNPs, (**E**–**H**) SEM images of nanoparticles at different scales, (**M**–**O**) Elemental mapping of RB-AgNPs showing scanned image of NPs with silver element distribution (pink), (**P**) EDX spectrum of the elemental mapped region showing highest peak for silver element. (**U**,**V**) TEM image of RB-AgNPs, (**W**,**X**) SAED pattern.

The purified and concentrated samples of RB-AuNPs and RB-AgNPs were studied by SEM, EDX, elemental mapping, TEM, and SEAD [23]. SEM image analysis clearly showed the maximum population of RB-AuNPs with spherical shape with very few triangles which cause polydispersities (Figure 3A–D). In contrast, for RB-AgNPs, we found a 100% population of nanoparticles as spherical and monodisperse (Figure 3E–H). There was no sign of any polydispersity. Next, we performed EDX, and elemental mapping of the selected region in the SEM scanned image of nanoparticles. The mapping results showed a clear map of gold and silver elements in RB-AuNPs (Figure 3I–K) and RB-AgNPs scanned images (Figure 3M–O). EDX also showed the highest and sharp peak for gold and silver elements, which confirmed the maximum distribution of gold (Figure 3L) and silver elements (Figure 3P) in respective samples, without any elemental contamination [29]. In addition, TEM analysis also revealed the spherical shape for RB-AuNPs and RB-AgNPs with a core diameter of 90–100 nm (Figure 3Q,R) and 20–30 nm (Figure 3U,V), respectively [22]. The SEAD pattern also corresponds to the crystalline nature of RB-AuNPs (Figure 3S,T) and RB-AgNPs (Figure 3W,X), in alignment with previous reports [24].

**Figure 4:**
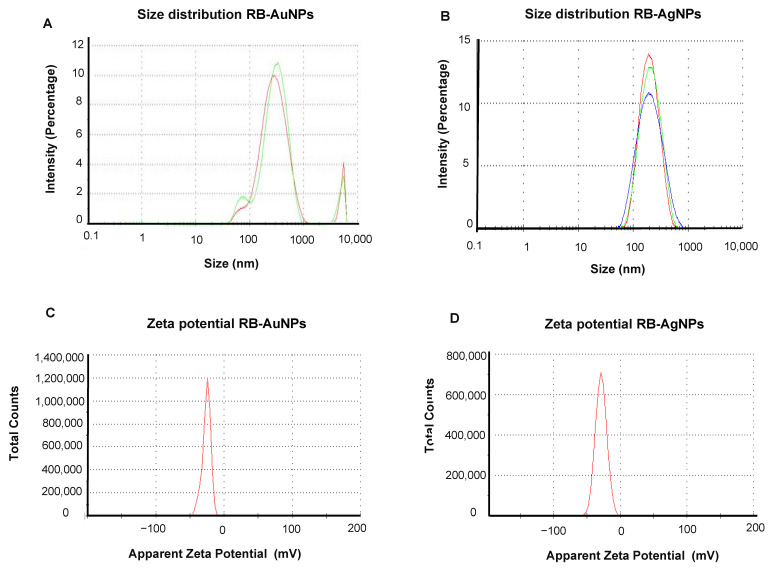
Dynamic light scattering analysis of RB-AuNPs and RB-AgNPs. (**A**) RB-AuNPs distribution concerning size and intensity (**B**) RB-AgNPs distribution concerning size and intensity. Different color represents technical replicates. (**C**) Zeta potential of RB-AuNPs and (**D**) RB-AgNPs, representing highly negative surface charge.

Next, we examined the nanoparticles with DLS to know the hydrodynamic diameter and zeta potential values. The results indicated that RB-AuNPs had a size of 280.1 nm (Figure 4A) with a polydispersity index (PDI) 0.512, and RB-AgNPs (Figure 4B) had a size of 177.1 nm with PDI 0.193 [25]. The zeta potential value of nanoparticles also indicated a highly negative surface charge for RB-AuNPs −25.6 mV (Figure 4C) and RB-AgNPs −28.8 mV (Figure 4D).

**Figure 5:**
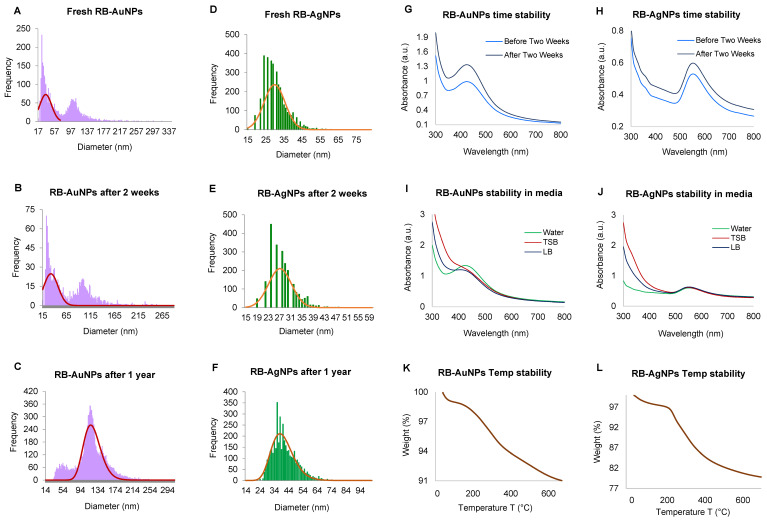
sp-ICPMS and stability analysis of RB-AuNPs and RB-AgNPs. ICPMS histogram of RB-AuNPs at different time intervals (**A**) fresh RB-AuNPs, (**B**) after two weeks, (**C**) after one year at 4 °C. ICPMS histogram of RB-AgNPs at different time intervals, (**D**) fresh RB-AuNPs, (**E**) after two weeks, (**F**) after one year at 4 °C. UV-Vis spectrum representing the stability analysis, before and after two weeks of incubation at RT for (**G**) RB-AuNPs and (**H**) RB-AgNPs; in a different medium (**I**) RB-AuNPs, (**J**) RB-AgNPs; at the temperature range from 20–700 °C measured by TGA instrument (**K**) RB-AuNPs, (**L**) RB-AgNPs.

The sp-ICPMS analysis for RB-AuNPs and RB-AgNPs was performed to know the concentration and yield of nanoparticles and their stability for different time intervals. The results showed that the concentration of RB-AuNPs was 0.67 µg/µL (Figure 5A), and for RB-AgNPs was 0.65 µg/µL (Figure 5D). The same samples were analyzed at a difference of two weeks (Figure 5B,E) and one year (Figure 5C,F). The obtained results indicated the same histogram without any fluctuations, which demonstrated that the formed nanoparticles remained in the same size and concentration for up to a year, i.e., highly stable. Stability tests were also conducted using UV-Vis spectrum analysis. The time difference scanned pattern also showed a similar and overlapping peak in a difference of two weeks for RB-AuNPs (Figure 5G) and RB-AgNPs (Figure 5H). Stability results of RB-AuNPs and RB-AgNPs in different media and aqueous solutions were also conducted, and the results demonstrated that the RB-AuNPs (Figure 5I) and RB-AgNPs (Figure 5J) remain stable in all three mediums but best in water. Temperature stability analysis showed complete degradation of nanoparticles concerning increasing temperature for RB-AuNPs (Figure 5K) and RB-AgNPs (Figure 5L).

**Figure 6:**
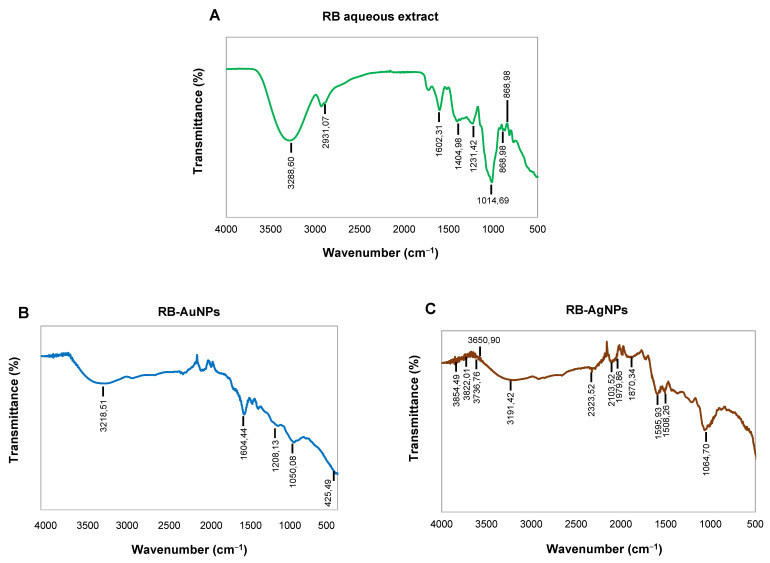
FT-IR spectrum of (**A**) freeze-dried aqueous extract of rowanberries and (**B**) RB-AuNPs, and (**C**) RB-AgNPs, which demonstrated the active surface groups for respective samples.

**Figure 7:**
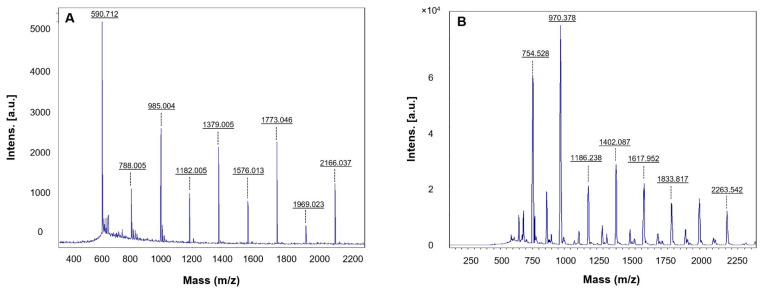
MALDI-TOF analysis of (**A**) RB-AuNPs and (**B**) RB-AgNPs demonstrated respective metal ions.

The FT-IR analysis of rowanberries extract confirmed the presence of many active groups (Figure 6A); for instance, the peak at 328,860 cm^−1^ corresponded to the –OH (hydroxyl group) of phenolic compounds and amine N–H/O–H stretch. The peak at 293,107 was the asymmetric stretching of a methyl group –CH_3_ and C–H stretching of alkanes or secondary amines [26]. The peak at 160,231 cm^−1^ represented –C=C stretching vibration in flavonoids and terpenoids and carbonyl group (–C=O) –stretching vibration of proteins or amide I, peak at 140,498 cm^−1^ to (N–H) stretching vibrations of proteins, peak at 123,142 to C–N aromatic amino groups, 101,469 peak to Carbonyl –C–O–C or –C–O stretching vibrations of amide linkages [30]. RB-AuNPs and RB-AgNPs showed overlapping peaks in similar regions, which indicated the presence of several active surface groups on nanoparticles surfaces originating from berries extract (Figure 6B,C) [31]. Finally, we performed the MALDI-TOF analysis to examine the protein content on the surface of the nanoparticles. The mass spectra showed a series of intense single peaks in the range between 590 and 3800 m/z (Figure 7A,B). For RB-AuNPs, several peaks could be assigned to gold ions, at 590.712, 788.005, 985.004, 1182.005, 1379.005, 1576.013, 1773.046, 1969.023, 2166.037 (Figure 7A). For RB-AgNPs, we detected several peaks assigned to silver ions of higher cationic species at 754.528, 970.378, 1186.238, 1402.087, 1617.952, 1833.817, and 2263.542 (Figure 7B) [4,21].

### 3.3. Antibacterial Activity of RB-AgNPs

**Figure 8:**
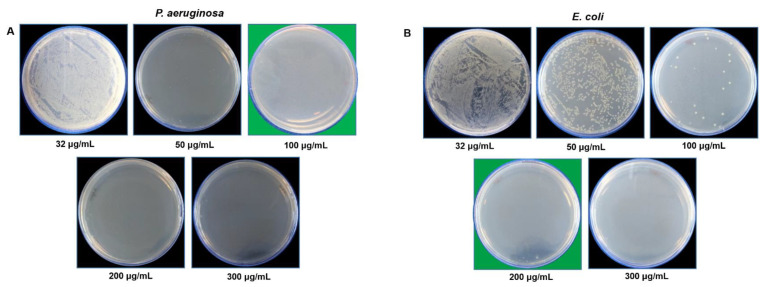
Cell viability test at different concentrations range from 32 to 300 µg/mL of RB-AgNPs for (**A**) *P. aeruginosa* and (**B**) *E. coli*. The green background shows the MBC values of respective pathogens with complete growth inhibition.

**Figure 9:**
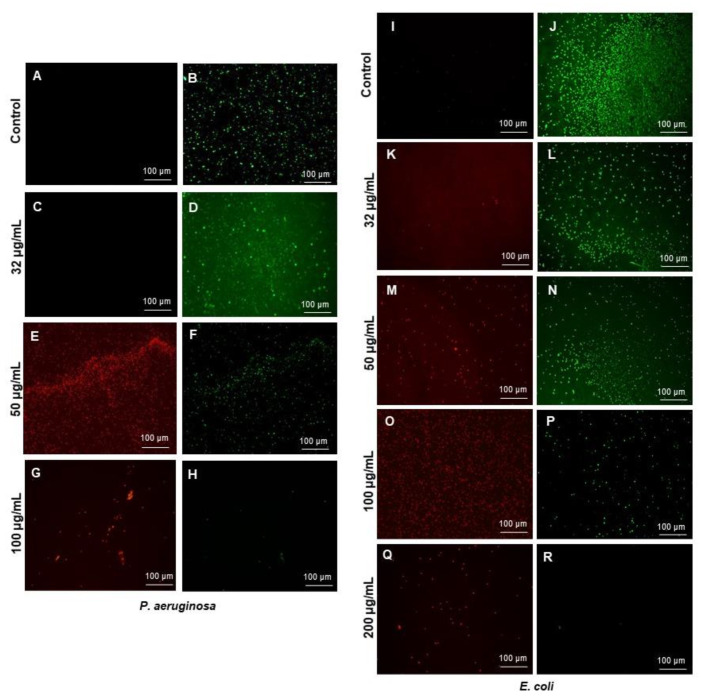
Live and dead staining of (**A**–**H**) *P. aeruginosa* and (**I**–**R**) *E. coli,* after treatment with RB-AgNPs at different concentrations. *P. aeruginosa* cells: (**A**,**B**) control without RB-AgNPs, (**C**,**D**) 32 µg/mL of RB-AgNPs, (**E**,**F**) 50 µg/mL of RB-AgNPs, (**G**,**H**) 100 µg/mL of RB-AgNPs. *E. coli* cells: (**I**,**J**) control without RB-AgNPs, (**K**,**L**) 32 µg/mL of RB-AgNPs, (**M**,**N**) 50 µg/mL of RB-AgNPs, (**O**,**P**) 100 µg/mL of RB-AgNPs, and (**Q**,**R**) 200 µg/mL of RB-AgNPs.

The RB-AgNPs produced in this study were further explored for the antibacterial activity against two Gram-negative pathogens, i.e., *P. aeruginosa* and *E. coli.* Figure 8 showed that RB-AgNPs completely killed the *P. aeruginosa* cells at 100 µg/mL and *E. coli* cells at 200 µg/mL. We further checked the viability by using live and dead staining kits. Cells exposed to different concentrations of RB-AgNPs were visualized under a fluorescent microscope after staining, and results are shown in Figure 9. Figure 9A–H for *P. aeruginosa* showed that most of the cells were viable at 32 µg/mL, and with increasing concentration, the green cells were not visible. At the same time, the red became dens, which means that the RB-AgNPs were toxic to cells and caused complete death of *P. aeruginosa* cells at 100 µg/mL.

**Figure 10:**
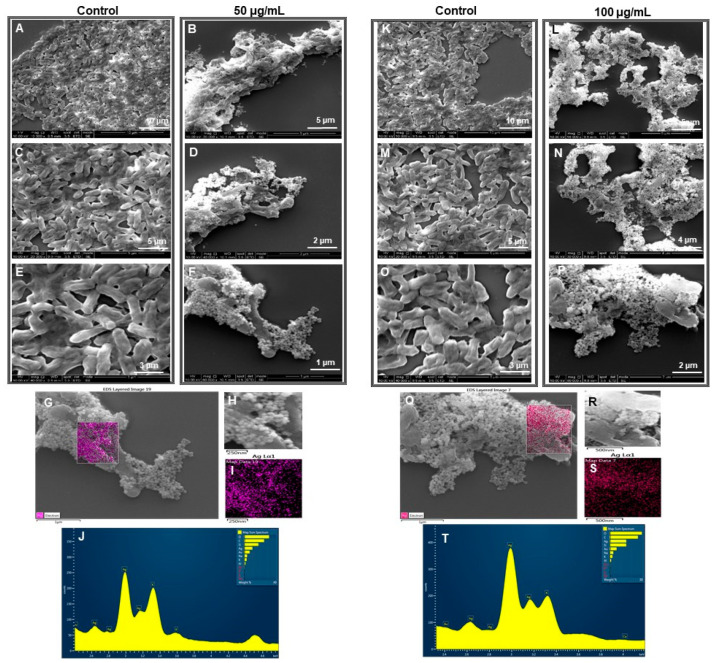
SEM analysis of *P. aeruginosa* cells after treatment with RB-AgNPs. (**A**–**F**) Control cells and RB-AgNPs treated cells with 50 µg/mL at different scales. (**G**) Scanned image of treated cells (**H**,**I**) elemental mapping of the selected area showing silver element in the treated cells, (**J**) EDX spectrum of the chosen area showing peak for silver element. (**K**–**P**) Control cells and RB-AgNPs treated cells with 100 µg/mL at different scales. (**Q**) Scanned image of treated cells (**R**,**S**) elemental mapping of the selected area showing silver element in the treated cells, (**T**) EDX spectrum of the chosen area showing peak for silver element.

**Figure 11:**
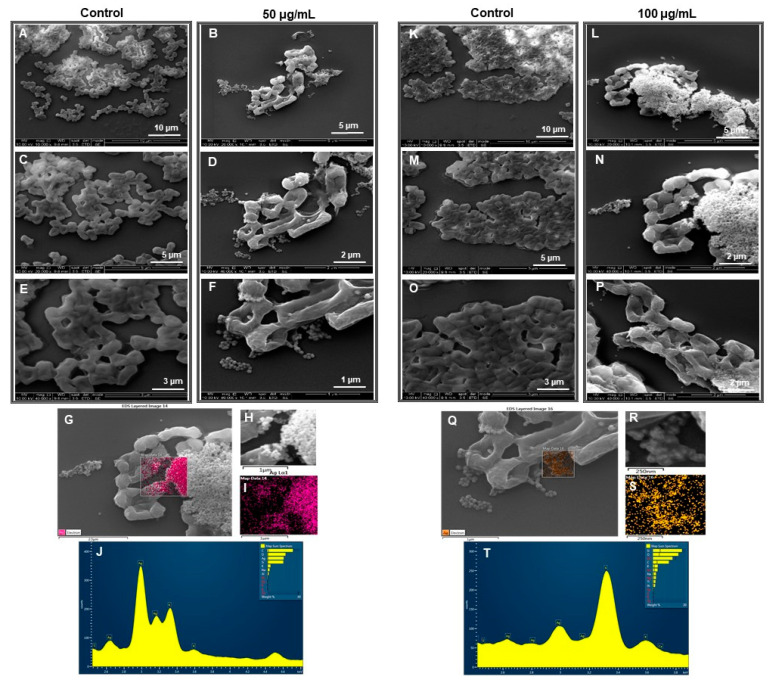
SEM analysis of *E. coli* cells after treatment with RB-AgNPs. (**A**–**F**) Control cells and RB-AgNPs treated cells with 50 µg/mL at different scales. (**G**) Scanned image of treated cells (**H**,**I**) elemental mapping of the selected area showing silver element in the treated cells, (**J**) EDX spectrum of the chosen area showing peak for silver element. (**K**–**P**) Control cells and RB-AgNPs treated cells with 100 µg/mL at different scales. (**Q**) Scanned image of treated cells (**R**,**S**) elemental mapping of the selected area showing silver element in the treated cells, (**T**) EDX spectrum of the chosen area showing peak for silver element.

Figure 9I–R, showed a complete red region of dead cells at 200 µg/mL concentration for *E. coli* cells. In addition, we also checked the morphology of the dead cells by using SEM [25]. SEM analysis of *P. aeruginosa* cells treated by RB-AgNPs was done at two different concentrations, i.e., 50 and 100 µg/mL. Figure 10A–F showed *P. aeruginosa* cells were completely covered with nanoparticles and open membrane and structures. The same behavior was found at 100 µg/mL concentration of RB-AgNPs, Figure 10K–P. The elemental mapping results also indicated that the damaged cells were completely covered with silver nanoparticles, Figure 10G–I,Q–S, which concluded that the cause of cell lysis was RB-AgNPs. In addition, EDX demonstrated the clear pic for silver element and Figure 10J,T, which showed the purity of RB-AgNPs in damaged cells. Figure 11 showed RB-AgNPs effects on *E. coli* cells at 50 µg/mL (Figure 11A–F) and 100 µg/mL (Figure 11K–P) concentrations of RB-AgNPs. The EDX and elemental mapping results indicated that the cells were covered predominantly with silver elements. Thus, the damage happened due to the silver ions released from RB-AgNPs Figure 11G–J,Q–T.

## 4. Discussion

Medicinal plants are quite popular for green nanoparticle production and stabilization, mostly resulting in monodisperse and stable nanoparticles [32,33]. These bio-reactions do not require additives because their resources or SM are adequately enriched with various biological components, which helps reduce and stabilize nanoparticles; these components are proteins, amino acids, sugars, polyphenols, flavonoids, terpenoids, polysaccharides, etc., [34]. Furthermore, the green SM usually stops the reaction after complete reduction to avoid the agglomeration of nanoparticles, hence promoting stability [33]. In the case of rowanberries, the active biological components documented were polyphenolic, primarily flavonoids and phenolic acids [6]. The extract of rowanberries reported many medicinal effects. For instance, aqueous extract of berries inhibited the growth of *E. faecalis*, *S. aureus*, and *S. enterica*, and the viability of *C. freundii* and *B. cereus*. Aqueous methanol extracts of rowanberries were potent antioxidants. The phenolic extracts of rowanberries had an inhibitory effect on the hemagglutination of *E. coli* HB101 (pRR7), which expresses the M hemagglutinin and delayed pathogenic *E. coli* growth. Acidified acetone extract of rowanberries demonstrated high antibacterial behavior against *S. enterica*, and *P. aeruginosa*, and weak activity towards the two *L. monocytogenes* strains and *P. vulgaris* [35]. Rowanberries contain proanthocyanidins, which reduce Caco-2 cell viability. Thus, due to the potential health benefits of rowanberries extract, we used it for the unexplored potential of reducing gold and silver salt to form RB-AuNPs and RB-AgNPs and compared them with various other fruit extracts also reported for similar action [36,37].

The primary observation of nanoparticles formation was supported by visual inspection of the color change in the SM and simultaneous recording of the UV-Vis spectra to check the SPR. UV-Vis techniques provide information about nanoparticles’ reduction, shape, size, and morphology [28]. RB-AuNPs showed maximum absorbance in the region of 500–600 nm and RB-AgNPs in 400–500 nm, consistent with the other reports. The kinetics of nanoparticles formation was monitored by recording the UV-Vis spectrum of SM at different extract-to-water ratios, temperature, time for synthesis, and salt concentrations. According to the spectral analysis for RB-AuNPs, the absorbance of the SPR band increased with an increasing ratio up to 2:8 (extract-to-water), and then there was a slight redshift in the wavelength. It was noticed that already the second-lowest amount of rowanberries aqueous extract was effective for the generation of RB-AuNPs. Further increase in the ratio of extract to water led to the peak broadening, which indicated nanoparticles size disturbance or polydispersity appearance [26]. According to the UV-Vis spectra of gold salt optimization studies, the absorption peak’s sharpness depended on the gold salt concentration used for synthesis, which further sharpened until 4 mM spectrum. It suggests that the nucleation and growth process occurred at 4 mM concentration within 5 s. Correlation between the peak sharpness and pictures of SM color was relatable and indicated the formation of RB-AuNPs.

RB-AgNPs optimizations studies indicated the highest peak in UV-Vis in the ratio of 6:4 (extract-to-water), which suggested that the complete reduction process occurred, and stable nanoparticles formed. At the lower ratios, the rate of nanoparticles formation was slow; hence, absorbance was also weaker. The reaction temperature also had significant effects on the size and morphology of the synthesized RB-AgNPs. Results of the UV-Vis spectrum showed the variation in the absorption spectra of RB-AgNPs synthesized at different temperatures, and the optimum peaks were obtained at 90 °C. There was no noticeable peak in the absorption spectra until 80 °C, indicating no formation of RB-AgNPs within that time. According to the literature, as the temperature rises, the kinetic energy of the molecules rises, and silver ions are used more quickly, leaving less room for particle size expansion. At a higher temperature, smaller nanoparticles with a uniform size distribution form [26]. The optimum reaction time found was 20 min; further, an increase in time resulted in nanoparticles agglomeration at 90 °C. Silver salt concentration for the optimum synthesis of RB-AgNPs appeared to be 4 mM; on increasing the concentration of silver salt further, it was found that the color of the solution turned dark brown, and peaks were almost overlapping with the 4 mM peak. Thus, the further formation of RB-AgNPs was restricted. In addition, the absorption peak got narrower with an increase in concentration.

The structural morphology of the nanoparticles was determined by using SEM and TEM techniques [38]. Obtained results showed that the nanoparticles were highly monodisperse. The shape is a very important aspect of nanoparticles to define their applications and interest. In addition, monodispersity or uniformity in shape is highly desirable, especially in green synthesis, which we achieved in the current study [39]. The uniformity of RB-AuNPs and RB-AgNPs directly corresponded to the SM and reaction parameters. The shape analysis from SEM and TEM showed spherical shapes for both RB-AuNPs and RB-AgNPs. For RB-AuNPs, the core particles range found was 90–100 nm, with uniformity in shape and size distribution, measured at a different scale [23]. RB-AuNPs also showed the clear distribution of gold elements in elemental mapping and EDX spectrum, which demonstrated the absence of any surface contamination.

For RB-AgNPs, the size distribution observed was 20–30 nm with a highly monodisperse spherical shape, clearly visible in SEM images [23]. Previously lead extract of rowanberries was reported to synthesize silver and gold nanoparticles within 15 min with an average size of 16 and 18 nm [40]. However, the nanoparticles showed polydispersity by appearing in different shapes, such as spherical, triangular, and hexagonal. In contrast, the current study demonstrated high monodispersity with spherical shapes. The elemental mapping also confirmed the consistent distributions for the silver element [41]. In the EDX graph, the presence of the elemental silver can be seen, which indicates the reduction of silver ions to elemental silver.

Another crucial factor to consider is the stability of the nanoparticles, which decides their long-term effects and application area. Nanoparticle stability varies depending on the synthesis technique, storage circumstances, and stabilizing and capping chemicals utilized throughout the process. [42]. In the current study, the change in absorption spectra of nanoparticles showed no major variation for the time and medium stability test, which indicates that the biomolecules present in the rowanberries extract act as a strong stabilizer. Based on FT-IR analysis, it was obvious that the generated nanoparticles were surrounded by many biological components, which provided stability [43]. In addition, FT-IR analysis revealed that the –OH of flavonoid compounds was responsible for the reduction and served as a corona layer around nanoparticles to provide stability. The –OH group can interact with the metal ions and reduce cationic silver (Ag^+^) ion to Ag^0^ or AgNPs. The stability was further confirmed by ICPMS, UV-Vis, and visible observation, which showed that the nanoparticles remained stable for more than a year when suspended in water and kept at 4 °C and for more than two weeks at room temperature. There was no sign of any precipitation or color change.

It has been noted that the antimicrobial activity or RB-AgNPs was very weak as compared to other reported silver nanoparticles formed from different berries. For instance, Masum et al. reported that the *Phyllanthus emblica* fruit extract mediated AgNPs activity against *Acidovorax oryzae* pathogen and found that 20 µg/mL concentration of AgNPs could affect bacterial growth, cell viability, biofilm formation, and swarming ability [44]. Other reports also available that aligned with our results; for instance, Patra et al. showed the MBC value of AgNPs synthesized from the outer peel of *Pisum sativum* was >100 µg/mL [43]. Likewise, RB-AgNPs could kill the complete cells of *P. aeruginosa* cells at 100 µg/mL and *E. coli* cells at 200 µg/mL. The SEM images showed cells membrane damage, with open-cell structures in both the pathogens, which displayed the cell lysis. Cell lysis by membrane damaging, ROS generation, proteins, and ribosomal inactivation are very common mechanisms reported for the antibacterial activity of AgNPs [45,46]. The reason behind the weak antibacterial activity of RB-AgNPs could be the formation of a thick and strong, biocompatible corona layer around the nanoparticles, which consisted of many plant residues [47]. The thickness of the corona layer was assumed based on the difference between the TEM size and DLS size of RB-AgNPs. TEM showed 20–30 nm core size, and DLS displayed 177 nm for hydrodynamic diameter [25]. There is always a difference between the core and hydrodynamic size. In the case of RB-AgNPs, we propose that this big difference corresponded to the capping layer surrounding the nanoparticles, which contributed to the DLS measurement but is likely to avoid detection in TEM due to high voltage. This thick corona layer provided stability and enhanced the biocompatibility of the formed RB-AgNPs. However, it may arguably reduce antibacterial activity. In addition to the stability tests conducted, the RB-AgNPs stability could also be observed in SEM images of treated cells. It was visible in the SEM images that after killing the cells, RB-AgNPs retained their spherical structure and size. The nanoparticles distribution was confirmed by EDX and elemental mapping, which showed that the maximum distribution was due to the silver element originating from RB-AgNPs. While the extremely thick corona layer might not favor antimicrobial applications, high biocompatibility and monodispersity make these nanoparticles potentially suitable in designing and developing novel and enhanced drug-delivery systems. These monodisperse nanoparticles can act as nanocarriers to deliver various therapeutic molecules for anticancer, anti-inflammatory, antioxidant, and antimicrobial activities, where overcoming the nanoparticle’s toxicity is the major challenge [13].

## 5. Conclusions

Gold and silver nanoparticles were successfully synthesized by a simple, rapid, efficient, and economical method using an aqueous extract of rowanberries. Extremely monodisperse, stable, and biocompatible nanoparticles were achieved by comparing the effects of temperature, reaction time, and reactants concentration. The results obtained using different characterization techniques showed that the biological components from rowanberries extract played a role in completely reducing and stabilizing nanoparticles. RB-AgNPs showed antimicrobial potential with an MBC value of 100 µg/mL against *P. aeruginosa* and 200 µg/mL against *E. coli.* However, more thorough research is needed to create practical and acceptable consumer applications for these nanoparticles in the medical field.

## Data Availability

Not applicable.
